# Supplementary figures and images for: Neuronal complexity is attenuated in preclinical models of migraine and restored by HDAC6 inhibition
Source: eLife. 2021 Apr 15;10:e63076. doi: 10.7554/eLife.63076 (PMC8147088; doi:10.7554/eLife.63076)

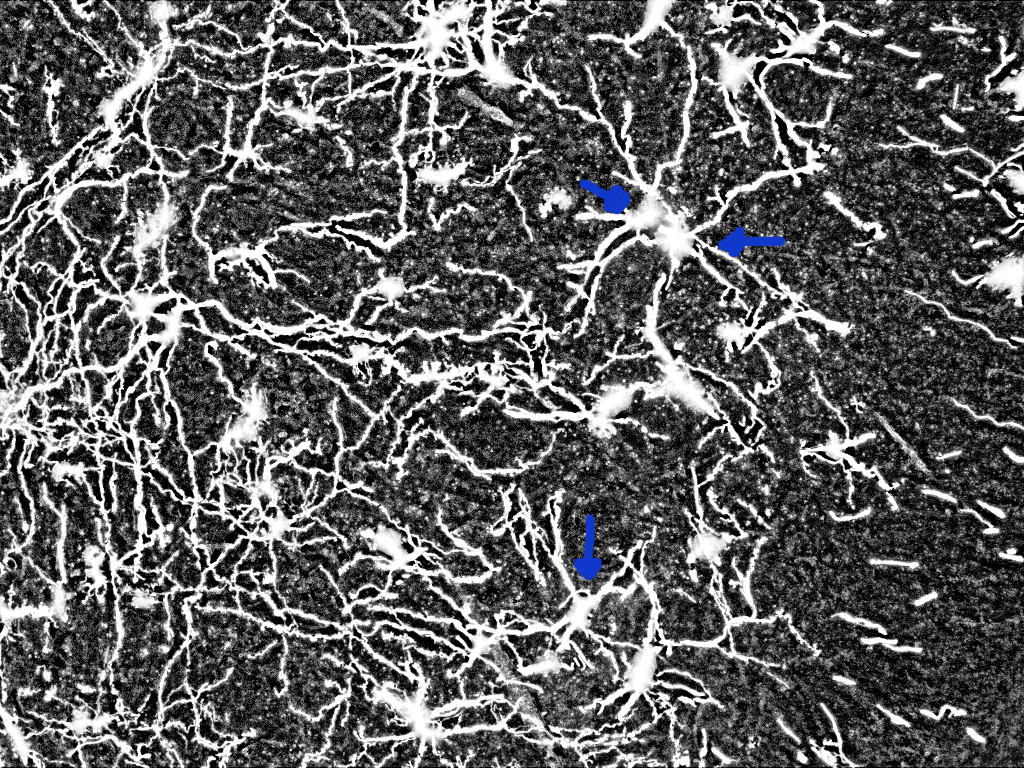

Supplement: Supplementary file 56 [file elife-63076-video1.gif]
