# Supplementary material for: Neuronal complexity is attenuated in preclinical models of migraine and restored by HDAC6 inhibition
Source: eLife. 2021 Apr 15;10:e63076. doi: 10.7554/eLife.63076 (PMC8147088; doi:10.7554/eLife.63076)
Supplement: Supplementary file 1. [file elife-63076-supp1.docx]

**Supplementary Table 1:** Summary of HDAC inhibition through ASV-85

| **Compound** | **Structure** | **HDAC1 ICSO (nM)** | **HDAC2 ICSO (nM)** | **HDAC3 ICSO (µM)** | **HDAC6 ICSO (µM)** |
| --- | --- | --- | --- | --- | --- |
| ASV-85 | 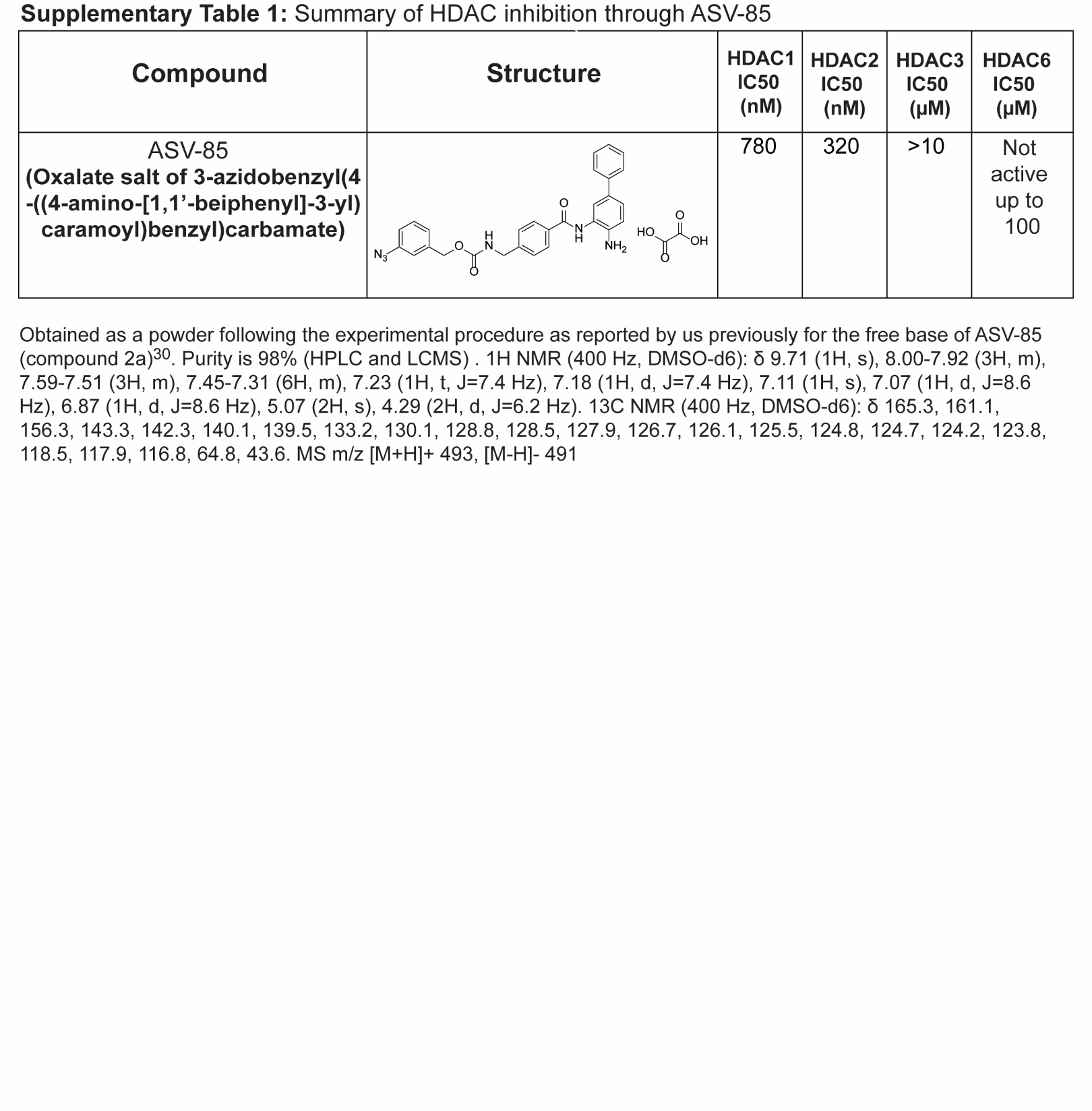 | 780 | 320 | >10 | Not |
| **(Oxalate salt of 3-azidobenzyl(4** |  |  |  |  | active |
| **-((4-amino-[1,1'-beiphenyl]-3-yl)** |  |  |  |  | up to |
| **caramoyl)benzyl)carbamate)** |  |  |  |  | 100 |

Obtained as a powder following the experimental procedure as reported by us previously for the free base of ASV-85 (compound 2a)30. Purity is 98% (HPLC and LCMS). 1H NMR (400 Hz, DMSO-d6): o 9.71 (1H, s), 8.00-7.92 (3H, m), 7.59-7.51 (3H, m), 7.45-7.31 (6H, m), 7.23 (1H, t, J=7.4 Hz), 7.18 (1H, d, J=7.4 Hz), 7.11 (1H, s), 7.07 (1H, d, J=8.6 Hz), 6.87 (1H, d, J=8.6 Hz), 5.07 (2H, s), 4.29 (2H, d, J=6.2 Hz). 13C NMR (400 Hz, DMSO-d6): o 165.3, 161.1,156.3, 143.3, 142.3, 140.1, 139.5, 133.2, 130.1, 128.8, 128.5, 127.9, 126.7, 126.1, 125.5, 124.8, 124.7, 124.2, 123.8, 118.5, 117.9, 116.8, 64.8, 43.6. MS m/z [M+H]+ 493, [M-H]- 491
