# Supplementary material for: Neuronal complexity is attenuated in preclinical models of migraine and restored by HDAC6 inhibition
Source: eLife. 2021 Apr 15;10:e63076. doi: 10.7554/eLife.63076 (PMC8147088; doi:10.7554/eLife.63076)
Supplement: Supplementary file 2. [file elife-63076-supp2.docx]

**Supplementary Table 2: Statistical Analysis**

**Two-Way ANOVA**

| **Figure** | Veh/NTG  Dfn,dfd F p 95%CI | | | | Time  Dfn,dfd F p | | | Interaction  Dfn,dfd F p | | |
| --- | --- | --- | --- | --- | --- | --- | --- | --- | --- | --- |
| 1B | 2,28 | 16.28 | <0.0001 | 0.3130, 0.6090 | 2, 28 | 19.16 | <0.0001 | 1, 14 | 44.62 | <0.0001 |
| 7A | 1, 22 | 19.16 | =0.0002 |  | 1.761, 38.75 | 14.25 | <0.0001 | 2, 44 | 14.05 | <0.0001 |

| **Figure** | Veh/NTG  Dfn,dfd F p 95% CI | | | | Veh/ACY  Dfn,dfd F p 95 % CI | | | | Interaction  Dfn,dfd F p 95% CI | | | | |
| --- | --- | --- | --- | --- | --- | --- | --- | --- | --- | --- | --- | --- | --- |
| 2B | 1, 86 | 7.891 | =0.0061 | 0.4864, 2.841 | 1, 86 | 14.61 | =0.0002 | -3.441, -1.086 | 1, 86 | 8.573 | =0.0044 | -5.815,-1.106 |  |
| 2C | 1, 170 | 0.5529 | =0.4581 | -65.99, 145.7 | 1, 170 | 109.8 | <0.0001 | -667.8,-456.1 | 1, 170 | 12.82 | =0.0004 | -595.8,-172.3 |  |
| 2F | 1, 25 | 0.07195 | =0.7907 | -5.063, 6.579 | 1 ,25 | 59.38 | <0.0001 | -27.60, -15.96 | 1, 25 | 8.537 | =0.0073 | -28.16,-4.874 |  |
| 3C | 1, 140 | 17.46 | <0.0001 | 1.024, 2.864 | 1, 140 | 3.424 | =0.0664 | -1.781, 0.0589 | 1, 140 | 5.701 | =0.0183 | -4.062,-0.3822 |  |
| 3D | 1, 140 | 1.741 | =0.1892 | -40.72, 204.1 | 1, 140 | 2.817 | =0.0955 | -226.4, 18.49 | 1, 140 | 7.717 | =0.0062 | -588.9, -99.19 |  |
| 3E | 1, 140 | 1.257 | =0.2641 | -2.141, 7.752 | 1, 140 | 2.347 | =0.1278 | -8.780, 1.113 | 1, 140 | 7.158 | =0.0084 | -23.28, -3.495 |  |
| 3F | 1, 140 | 46.33 | <0.0001 | 2.138, 3.889 | 1, 140 | 0.5204 | =0.4719 | -0.556, 1.195 | 1, 140 | 0.2214 | =0.6387 | -2.168, 1.334 |  |
| 3G | 1, 140 | 17.41 | <0.0001 | 39.94, 111.9 | 1, 140 | 0.2259 | =0.6353 | -44.61, 27.32 | 1, 140 | 0.0002424 | =0.9876 | -72.50, 71.37 |  |
| 3H | 1, 140 | 14.99 | =0.0002 | 3.725, 11.50 | 1, 140 | 0.4044 | =0.5258 | -5.136, 2.636 | 1, 140 | 0.007190 | =0.9325 | -7.439, 8.105 |  |

| **Figure** | Sham/CSD  Dfn,dfd F p 95%CI | | | | Veh/ACY  Dfn,dfd F p 95%CI | | | | Interaction  Dfn,dfd F p 95%CI | | | |
| --- | --- | --- | --- | --- | --- | --- | --- | --- | --- | --- | --- | --- |
| 6C | 1, 224 | 27.80 | <0.0001 | 2.274, 4.989 | 1, 224 | 60.66 | <0.0001 | -6.722, -4.007 | 1, 224 | 0.05986 | =0.8069 | -3.052, 2.378 |
| 6D | 1, 224 | 16.48 | <0.0001 | 149.1, 430.4 | 1, 224 | 25.18 | <0.0001 | -498.8, -217.5 | 1, 224 | 1.506 | =0.2211 | -456.5, 106.1 |
| 6G | 1, 224 | 10.87 | =0.0011 | 3.601, 14.30 | 1, 224 | 23.98 | <0.0001 | -18.64, -7.944 | 1, 224 | 2.098 | =0.1489 | -18.56, 2.834 |

| **Figure** | Veh/NTG  Dfn,dfd F p 95%CI | | | | Veh/Olcegepant  Dfn,dfd F p 95%CI | | | | Interaction  Dfn,dfd F p 95%CI | | | |
| --- | --- | --- | --- | --- | --- | --- | --- | --- | --- | --- | --- | --- |
| 7D | 1, 140 | 26.82 | <0.0001 | 1.477, 3.301 | 1, 140 | 5.237 | =0.0236 | -1.967, -0.1437 | 1, 140 | 4.193 | =0.0425 | -3.713, -0.0651 |
| 7E | 1, 140 | 7.541 | =0.0068 | 35.59, 218.6 | 1, 140 | 6.853 | =0.0098 | -212.6, -29.65 | 1, 140 | 9.009 | =0.0032 | -460.8, -94.82 |
| 7H | 1, 140 | 5.447 | =0.0210 | 0.6772, 8.184 | 1, 140 | 6.371 | =0.0127 | -8.545, -1.038 | 1, 140 | 6.151 | =0.0143 | -16.92, -1.910 |

| **Figure** | Veh/NTG  Dfn,dfd F p 95%CI | | | | Veh/ACY738  Dfn,dfd F p 95%CI | | | | Interaction  Dfn,dfd F p 95%CI | | | |
| --- | --- | --- | --- | --- | --- | --- | --- | --- | --- | --- | --- | --- |
| Supp 2A | 1, 32 | 1.130 | =0.2958 | -0.2221, 0.7067 | 1, 32 | 23.09 | <0.0001 | -1.560, -0.6312 | 1, 32 | 0.3248 | =0.5727 | -0.6689, 1.189 |
| Supp 2B | 1, 32 | 5.882 | =0.0211 | -0.8484, -0.07385 | 1, 32 | 38.63 | <0.0001 | -1.569, -0.7943 | 1, 32 | 6.986 | =0.0126 | -1.780, -0.2305 |
| Supp 2C | 1, 28 | 0.6118 | =0.4407 | -1.244, 0.5567 | 1, 28 | 37.92 | <0.0001 | -3.608, -1.807 | 1, 28 | 1.112 | =0.3008 | -2.728,0.8741 |
| Supp 2D | 1, 16 | 0.003661 | =0.9525 | -0.1741, 0.1645 | 1, 16 | 4.649 | =0.0466 | -0.3415, -0.002903 | 1, 16 | 0.0185 | =0.9025 | -0.3584, 0.3187 |
| Supp 2E | 1, 20 | 0.06052 | =0.4457 | -0.1302, 0.05946 | 1, 20 | 9.357 | =0.0062 | -0.2339, -0.04423 | 1, 20 | 0.2371 | =0.6316 | -0.1454, 0.2339 |

**Unpaired t-test**

| **Figure** | F test to compare variances  dfn, dfd F P | | | Unpaired t Test  P T df | | | Mean Veh | Mean NTG | SEM | 95% Confidence Interval |
| --- | --- | --- | --- | --- | --- | --- | --- | --- | --- | --- |
| 1E | 35, 35 | 1.073 | 0.8354 | 0.0001 | 4.029 | 70 | 7.806 | 5.889 | 0.4758 | -2.866, -0.9678 |
| 1F | 35, 35 | 1.197 | 0.5972 | 0.0066 | 2.798 | 70 | 262.6 | 208.3 | 19.41 | -93.04, -15.60 |
| 1I | 35, 35 | 1.202 | 0.589 | 0.0193 | 2.396 | 70 | 29.56 | 24.19 | 2.238 | -9.825, -0.8977 |
| 5D | 6, 6 | 3.050 | 0.2006 | 0.0002 | 5.307 | 12 | 13.86 | 8.286 | 1.050 | -7.859, -3.284 |
| Supp 1B | 35, 35 | 1.106 | 0.7664 | <0.0001 | 6.486 | 70 | 20.69 | 15.28 | 0.8352 | -7.082, -3.751 |
| Supp 1C | 35, 35 | 1.002 | 0.9943 | <0.0001 | 4.428 | 70 | 613.4 | 457.2 | 35.27 | -226.5, -85.83 |
| Supp 1D | 35, 35 | 1.082 | 0.8174 | <0.0001 | 4.127 | 70 | 64.08 | 48.36 | 3.810 | -23.32, -8.124 |
| Supp 1F | 35, 35 | 1.108 | 0.7626 | <0.0001 | 4.208 | 70 | 13.69 | 10.31 | 0.8054 | -4.995, -1.783 |
| Supp 1G | 35, 35 | 1.167 | 0.6506 | 0.0089 | 2.693 | 70 | 535.3 | 417.9 | 43.63 | -204.5, -30.48 |
| Supp 1H | 35, 35 | 1.265 | 0.4911 | 0.0101 | 2.645 | 70 | 57.03 | 44.67 | 4.674 | -21.68, -3.040 |
| Supp 1J | 29, 35 | 2.231 | 0.0244 | 0.8531 | 0.1860 | 64 | 17.6 | 17.33 | 1.434 | -3.131, 2.598 |
| Supp 1K | 29, 35 | 2.246 | 0.0232 | 0.9467 | 0.06712 | 64 | 515.5 | 519.3 | 57.20 | -110.4, 118.1 |
| Supp 1L | 29, 35 | 2.136 | 0.0331 | 0.9106 | 0.1128 | 64 | 53.23 | 53.89 | 5.813 | -10.96, 12.27 |
| Supp 1N | 35, 35 | 1.326 | 0.4077 | 0.2788 | 1.092 | 70 | 10.72 | 11.42 | 0.6362 | -0.5744, 1.963 |
| Supp 1O | 35, 35 | 1.151 | 0.6805 | 0.5715 | 0.5685 | 70 | 413.1 | 394.2 | 33.18 | -85.04, 47.31 |
| Supp 1P | 35, 35 | 1.226 | 0.5507 | 0.9016 | 0.1241 | 70 | 43.53 | 43.11 | 3.357 | -7.111, 6.278 |
| Supp 1R | 35, 35 | 1.217 | 0.5646 | 0.6882 | 0.4030 | 70 | 9.833 | 9.639 | 0.4825 | -1.157, 0.7678 |
| Supp 1S | 35, 35 | 1.076 | 0.8299 | 0.7402 | 0.3329 | 70 | 331.9 | 323.8 | 24.17 | -56.24, 40.15 |
| Supp 1T | 35, 35 | 1.001 | 0.9985 | 0.7103 | 0.3730 | 70 | 37.00 | 35.97 | 2.756 | -6.524, 4.468 |
| Supp 3C | 9, 9 | 1.690 | 0.4464 | 0.0128 | 2.765 | 18 | 1.126 | 1.951 | 0.2984 | 0.1982, 1.452 |
| Supp 4A | 35, 35 | 2.413 | 0.0109 | 0.004 | 2.980 | 70 | 19.75 | 14.83 | 1.650 | -8.207, -1.626 |
| Supp 4B | 35, 35 | 1.912 | 0.0592 | 0.334 | 0.9729 | 70 | 493.4 | 441 | 53.85 | -159.8, 55.01 |
| Supp 4C | 35, 35 | 1.655 | 0.1412 | 0.5069 | 0.6671 | 70 | 49.72 | 46.17 | 5.329 | -14.18, 7.074 |

**Three-Way ANOVA**

| **Figure** | Time  Dfn,dfd F p | | | Veh-NTG  Dfn,dfd F p | | | Veh-ACY  Dfn,dfd F p | | | Time Veh-NTG  Dfn,dfd F p | | | Time Veh-ACY  Dfn,dfd F p | | | Veh-NTG Veh-ACY  Dfn,dfd F p | | | Interaction  Dfn,dfd F p | | |
| --- | --- | --- | --- | --- | --- | --- | --- | --- | --- | --- | --- | --- | --- | --- | --- | --- | --- | --- | --- | --- | --- |
| 3A | 3, 35 | 5.174 | =0.0046 | 1, 35 | 209.8 | <0.0001 | 1, 35 | 16.85 | =0.0002 | 3, 35 | 5.524 | =0.0033 | 3, 35 | 6.579 | =0.0012 | 1, 35 | 33.20 | <0.0001 | 3, 35 | 11.19 | <0.0001 |

| Figure | Time  Dfn,dfd F p | | | Veh-NTG  Dfn,dfd F p | | | Veh-TSA  Dfn,dfd F p | | | Time Veh-NTG  Dfn,dfd F p | | | Time Veh-TSA  Dfn,dfd F p | | | Veh-NTG Veh-TSA  Dfn,dfd F p | | | Interaction  Dfn,dfd F p | | |
| --- | --- | --- | --- | --- | --- | --- | --- | --- | --- | --- | --- | --- | --- | --- | --- | --- | --- | --- | --- | --- | --- |
| 4A | 1, 20 | 22.30 | =0.0001 | 1, 20 | 271.6 | <0.0001 | 1, 20 | 9.193 | =0.0066 | 1, 20 | 0.2415 | =0.6285 | 1, 20 | 5.581 | =0.0284 | 1, 20 | 13.17 | =0.0017 | 1, 20 | 8.688 | =0.0080 |

| Figure | Time  Dfn,dfd F p | | | Veh-NTG  Dfn,dfd F p | | | Veh-RN-73 Low  Dfn,dfd F p | | | Time Veh-NTG  Dfn,dfd F p | | | Time Veh- RN-73 Low  Dfn,dfd F p | | | Veh-NTG Veh- RN-73 Low  Dfn,dfd F p | | | Interaction  Dfn,dfd F p | | |
| --- | --- | --- | --- | --- | --- | --- | --- | --- | --- | --- | --- | --- | --- | --- | --- | --- | --- | --- | --- | --- | --- |
| 4B | 3, 84 | 5.183 | =0.0025 | 1, 28 | 240.9 | <0.0001 | 1, 28 | 3.347 | =0.0780 | 3, 84 | 8.208 | <0.0001 | 3, 84 | 3.241 | =0.0261 | 1, 28 | 4.629 | =0.0402 | 3, 84 | 7.214 | =0.0002 |

| Figure | Time  Dfn,dfd F p | | | Veh-NTG  Dfn,dfd F p | | | Veh-RN-73 High  Dfn,dfd F p | | | Time Veh-NTG  Dfn,dfd F p | | | Time Veh- RN-73 High  Dfn,dfd F p | | | Veh-NTG Veh- RN-73 High  Dfn,dfd F p | | | Interaction  Dfn,dfd F p | | |
| --- | --- | --- | --- | --- | --- | --- | --- | --- | --- | --- | --- | --- | --- | --- | --- | --- | --- | --- | --- | --- | --- |
| 4B | 3, 84 | 15.01 | <0.0001 | 1, 28 | 135.3 | <0.0001 | 1, 28 | 13.87 | =0.0009 | 3, 84 | 10.61 | <0.0001 | 3, 84 | 15.52 | <0.0001 | 1, 28 | 7.258 | =0.0118 | 3, 84 | 7.396 | =0.0002 |

| Figure | Time  Dfn,dfd F p | | | Veh-NTG  Dfn,dfd F p | | | Veh-ASV  Dfn,dfd F p | | | Time Veh-NTG  Dfn,dfd F p | | | Time Veh- ASV  Dfn,dfd F p | | | Veh-NTG Veh-ASV  Dfn,dfd F p | | | Interaction  Dfn,dfd F p | | |
| --- | --- | --- | --- | --- | --- | --- | --- | --- | --- | --- | --- | --- | --- | --- | --- | --- | --- | --- | --- | --- | --- |
| 4C | 1.946, 38.93 | 2.566 | =0.0912 | 1, 20 | 118.1 | <0.0001 | 1, 20 | 8.327e-005 | =0.9928 | 2, 40 | 3.359 | =0.0448 | 2, 40 | 0.9971 | =0.3779 | 1, 20 | 0.0021 | =0.9643 | 2, 40 | 0.9580 | =0.3923 |

| Figure | Time  Dfn,dfd F p | | | Veh-NTG  Dfn,dfd F p | | | Veh-ACY  Dfn,dfd F p | | | Time Veh-NTG  Dfn,dfd F p | | | Time Veh- ACY  Dfn,dfd F p | | | Veh-NTG Veh-ACY  Dfn,dfd F p | | | Interaction  Dfn,dfd F p | | |
| --- | --- | --- | --- | --- | --- | --- | --- | --- | --- | --- | --- | --- | --- | --- | --- | --- | --- | --- | --- | --- | --- |
| 4D | 2, 15 | 6.947 | =0.0073 | 1, 15 | 5.672 | =0.0309 | 1, 15 | 3.922 | =0.663 | 2, 15 | 1.017 | =0.3854 | 2, 15 | 1.218 | =0.3234 | 1, 15 | 6.167 | =0.0253 | 2, 15 | 1.870 | =0.1884 |

| Figure | Time  Dfn,dfd F p | | | Veh-NTG  Dfn,dfd F p | | | Veh-Olcegepant  Dfn,dfd F p | | | Time Veh-NTG  Dfn,dfd F p | | | Time Veh-Olcegepant  Dfn,dfd F p | | | Veh-NTG Veh-Olcegepant  Dfn,dfd F p | | | Interaction  Dfn,dfd F p | | |
| --- | --- | --- | --- | --- | --- | --- | --- | --- | --- | --- | --- | --- | --- | --- | --- | --- | --- | --- | --- | --- | --- |
| 7B | 1, 10 | 8.126 | =0.0172 | 1, 10 | 32.12 | =0.0002 | 1, 10 | 19.29 | =0.0014 | 1, 10 | 3.490 | =0.0913 | 1, 10 | 23.40 | =0.0007 | 1, 10 | 3.993 | =0.0736 | 1, 10 | 4.802 | =0.0532 |
